# Novel At-Home Mother’s Milk Conductivity Sensing Technology as an Identification System of Delay in Milk Secretory Activation Progress and Early Breastfeeding Problems: Feasibility Assessment

**DOI:** 10.2196/43837

**Published:** 2023-08-22

**Authors:** Sharon Haramati, Anastasia Firsow, Daniela Abigail Navarro, Ravid Shechter

**Affiliations:** 1 MyMilk Laboratories Ltd Glil Yam Israel; 2 Department of Nutrition Sciences Ariel University Ariel Israel

**Keywords:** breastfeeding, feasibility, human milk, biomarker, remote sensing technology, mobile health, retrospective, secretory activation, lactogenesis, milk supply, milk, sensing technology, monitoring tool, lactation, exclusive breastfeeding, breastfeed, maternal health, maternal and infant health, infant health, maternal and child health, prolactin, lactation consultant, lactation support provider, mother, milk maturation

## Abstract

**Background:**

Prolonged exclusive breastfeeding is a public health priority and a personal desire by mothers; however, rates are low with milk supply challenges as a predominant cause. Early breastfeeding management at home is key. Milk electrolytes, mainly sodium ions, are accepted as biomarkers of secretory activation processes throughout the first weeks after birth and predictors for prolonged breastfeeding success, although they are not incorporated into routine care practice.

**Objective:**

The aim of this study was to test the feasibility of a novel handheld smartphone-operated milk conductivity sensing system that was designed to compute a novel parameter, milk maturation percent (MM%), calculated from milk sample conductivity for tracking individual secretory activation progress in a real-world home setting.

**Methods:**

System performance was initially evaluated in data collected from laboratory-based milk analysis, followed by a retrospective analysis of observational real-world data gathered with the system, on the spot in an at-home setting, implemented by lactation support providers or directly by mothers (N=592). Data collected included milk sample sensing data, baby age, and self-reported breastfeeding status and breastfeeding-related conditions. The data were retroactively classified in a day after birth–dependent manner. Results were compared between groups classified according to breastfeeding exclusivity and breastfeeding problems associated with ineffective breastfeeding and low milk supply.

**Results:**

Laboratory analysis in a set of breast milk samples demonstrated a strong correlation between the system’s results and sodium ion levels. In the real-world data set, a total of 1511 milk sensing records were obtained on the spot with over 592 real-world mothers. Data gathered with the system revealed a typical time-dependent increase in the milk maturation parameter (MM%), characterized by an initial steep increase, followed by a moderate increase, and reaching a plateau during the first weeks postpartum. Additionally, MM% levels captured by the system were found to be sensitive to breastfeeding status classifications of exclusive breastfeeding and breastfeeding problems, manifested by differences in group means in the several-day range after birth, predominantly during the first weeks postpartum. Differences could also be demonstrated for the per-case time after birth–dependent progress in individual mothers.

**Conclusions:**

This feasibility study demonstrates that the use of smart milk conductivity sensing technology can provide a robust, objective measure of individual breastfeeding efficiency, facilitating remote data collection within a home setting. This system holds considerable potential to augment both self-monitoring and remote breastfeeding management capabilities, as well as to refine clinical classifications. To further validate the clinical relevance and potential of this home milk monitoring tool, future controlled clinical studies are necessary, which will provide insights into its impact on user and care provider satisfaction and its potential to meet breastfeeding success goals.

## Introduction

Despite the growing body of knowledge regarding breastfeeding benefits for both the mother and baby [1,2] and efforts of national and international health organizations to promote and support breastfeeding, exclusive breastfeeding rates remain low [3]. While breastfeeding initiation rates are relatively high (>80%), use of commercial milk formula (hereafter referred to as “formula”) is high [4]. There is a sharp drop where a third of mothers stop breastfeeding within the first month after birth [5,6], with insufficient milk supply being the most prevalent self-reported contributing factor [4,7].

Postglandular (or secondary) lactation insufficiency, caused by ineffective or infrequent milk removal, is reportedly the most common cause of inability to support an exclusively breastfed infant’s optimal growth and development [8]. Early proactive and corrective care, when the lactation process is most amenable to positive manipulation, can have a great impact on milk supply in the short and long term [4,9].

Lactogenesis, the process by which the mammary glands develop and regulate milk secretion, is a complex physiological process involving an orchestrated series of endocrine and autocrine/paracrine hormonal changes. The secretory activation stage (known as lactogenesis II) is tightly related to effective breastfeeding with frequent and adequate milk removal from the breast [10]; therefore, this stage can be dramatically perturbed by the introduction of nonmother’s milk feeds [4]. Inadequate secretory activation progress constitutes a higher risk for insufficient subsequent milk production, leading to excessive infant weight loss and early exclusive and/or any breastfeeding cessation [11,12]. Thus, objective assessment tools for monitoring an individual’s lactogenesis progress warrant attention.

Researchers over the past 70 years have identified a variety of components in human milk indicative of mammary gland progress toward the production of copious mature milk, including electrolytes such as sodium, potassium, and chloride ions, alongside lactose, protein, and citrate [13-18]. Electrolytes, mainly sodium ions, and related measures, including milk conductivity, show unique dynamics over the first weeks postpartum. These factors have been demonstrated to serve as useful biomarkers for successful or failed secretory activation, as a potential measure of increased risk for early breastfeeding cessation [19-24], and were recently assessed as a potential care support tool [25-28]. Early and frequent breast stimulation and efficient emptying were shown to improve marker dynamics and milk supply. Milk electrolyte balance was also shown to be affected by breast inflammation, a well-reported obstacle to breastfeeding success [29,30]. However, the main testing methods available for these biomarkers to date require laboratory-based approaches, hindering their large-scale practical use.

Breastfeeding is managed by the mother at home. Early education and support by lactation consultants via home visits or remotely via telehealth, especially in the first 4 weeks after birth, were shown to impact the success and maintenance of exclusive breastfeeding [31-34]. In recent years, mobile health apps have been paving the way toward more personalized self-care. Breastfeeding tracking and support apps have increasingly been adopted both in nonclinical and clinical contexts [35]. Augmenting health tracking tools with sensor technologies operated in the home setting has the potential to advance our understanding of the dynamics of breastfeeding physiology and low supply etiologies, demonstrating potential for future clinical care implementation.

We here describe the development and early real-world evidence from the use of a novel milk conductivity sensing system designed for tracking individual mothers’ milk secretory activation progress at home. The handheld milk conductivity sensing device and a mobile app were designed for an instant computation of a milk maturation percent (MM%) parameter that is computed from milk sample conductivity measured by the device, reflecting sample maturation status in percentages within the full dynamic range from initial colostrum to fully mature milk; the lower the MM%, the less advanced the mother is in her lactogenesis phase progress and supply process.

The aim of this study was to test the feasibility of this smartphone-operated milk conductivity sensing system to track individual secretory activation progress in a real-world home setting and its ability to identify different breastfeeding classifications associated with milk supply in a diverse set of real-world users.

## Methods

### Research Design

This retrospective study was based on a data set gathered by real-world users of a novel milk conductivity sensing system with the purpose of assessing individual secretory activation progress at home. The study objective was to assess feasibility of the novel system for remotely tracking secretory activation status at home. We referred to the STROBE (Strengthening the Reporting of Observational Studies in Epidemiology) guidelines for reporting cross-sectional observational data and also to the Guidelines and Checklist for the Reporting on Digital Health Implementations, when applicable.

System performance was initially evaluated in data collected from laboratory-based milk analysis, followed by a retrospective analysis of observational real-world data gathered with the system implemented by lactation support providers and directly by mothers in the home setting.

The available data set in the company’s database allowed for a cost-effective analysis in studying the relationship between milk maturation progress and breastfeeding exclusivity and related problems, which can provide insight for the design of future prospective and randomized trials.

Users joined the technology assessment on their sole discretion; downloaded the app; and consented before use to terms of use, privacy policy, and data usage for anonymous research purposes (see Multimedia Appendix 1). Users’ data were adequately secured according to the organization’s privacy policies designed for maintaining confidentiality and privacy, and the study data set was deidentified at extraction for analysis.

The system was not intended for diagnosing or treating a medical condition but rather as an informational/educational, noninvasive, and low-risk tool for promoting a healthy lifestyle. This was clearly indicated at all steps of registration. The system was implemented in a nonclinical nonacademic setting; any and all system interaction was at the sole discretion of users.

### Ethical Approval

This study was approved by the Institutional Ethics Committee of Ariel University (approval number AU-HEA-AN-20221220).

### Setting and Context

This retrospective analysis observed data records from breastfeeding mothers in the state of Israel. According to formal figures published by the Israel Ministry of Health, the breastfeeding initiation rate in Israel is 93% and exclusive breastfeeding rates drop to 55% by 1 month and to 20% at 6 months (data collected by the software “Healthy thinking” within “Tipat Halav” national well-baby visit centers [36]).

### Sample

The study population included users registered between July 2018 and October 2020. A total of 592 mothers were included in the retrospective analysis (555 recorded by lactation support providers and 37 self-recorded directly). Users were not compensated for participation. Several users covered shipment costs. Sample demographics, including data on birth weight, gestational week at birth, parity, age, sex of the infant, and more, can be found in Multimedia Appendix 2. The study set was not controlled and was subjected to selection bias, and therefore cannot and is not intended to represent the general population.

### Measurement Apparatus and Software

The milk conductivity sensing system comprised a handheld device for sensing the conductivity of small human milk samples and a smartphone app for data recording, computation of MM%, and user output.

The device included a sample cell for holding a small milk sample with two fixed electrodes (cell constant K=1) and a temperature probe. The electronic board featured a microprocessor, LCD display, and was powered by two 1.5-V batteries. Sample conductivity was computed from measured conductance, corrected to 25°C, and calibrated per device using potassium chloride standard solutions. The device’s laboratory performance was tested for linearity, precision, accuracy, repeatability, and stability using standard solutions and frozen breast milk specimens (see Multimedia Appendix 2), and was compared with a lab-grade conductivity analyzer (LAQUAtwin, EC-33 HORIBA).

Several milk sample cell configurations were tested to minimize sample size. Comparable results were observed for sample volumes of 0.2 ml, 0.5 ml, 1 ml, and 2 ml, leading to the selection of minimal volume cells of 0.5 ml and 0.2 ml for device assembly (see Multimedia Appendix 2 [37]).

Electrical conductivity, a measure of a fluid’s current conduction capability, is linked to the concentration and mobility of ions. The charged particles in body fluids are primarily sodium, potassium, and chloride. The relationship between breast milk conductivity and specific electrolyte concentrations has been previously reported [17].

We investigated sodium ion (Na^+^), a well-studied breast milk electrolyte linked to lactogenesis [14,15,38] and lactation adequacy [19,22,23], in relation to the measurements obtained with our apparatus. We used a large set of breast milk samples (n=79) for this lab-based test, plotting the measured relative conductivity against the sodium concentration, as assessed by a lab-grade ion-selective electrode analyzer (Roche/Hitachi Cobas 6000 c501 system, ISE module) and a portable Na^+^-selective electrode instrument (LAQUATwin, NA-11 HORIBA) that was previously validated for human milk [37]. The results showed a strong correlation, suggesting Na^+^ ion as a key factor in milk conductivity (see Multimedia Appendix 2 [15,29]). No correlation was found with Na^+^ or conductivity and certain milk components demonstrating significant interindividual variability (fat; vitamins A, B1, B2, B6, and B12; and caffeine; for details see Multimedia Appendix 2 [39]). However, protein levels, which show similar dynamics to Na^+^ in lactogenesis [15] and little interindividual difference in mature milk, positively correlated with both conductivity and Na^+^. Postpartum day-specific conductivity patterns mirrored Na^+^ trends, similar to the results reported by Neville et al [15], and mirrored Na^+^ trends in certain inflammation-associated breast pain cases, confirming previous reports [29,30] (see Multimedia Appendix 2 [19,22,23]).

MM% was calculated from device-measured conductivity using a predefined equation, based on an empirical data set of 625 breast milk samples from the first 10 days postpartum (detailed in Multimedia Appendix 2). The result was computed for each breast, saved, and displayed on the user interfaces in seconds.

Two apps were developed: a user-friendly app for mothers to record and track daily inputs, complemented by educational materials (iOS-compatible; see Multimedia Appendix 2) and a separate app for lactation support providers to manage records of different mothers (iOS- and Android-compatible; Multimedia Appendix 2).

System-recorded data included the baby’s birth date and time, birth weight, sex, breastfeeding exclusivity, and milk sample measurements. Users could self-report additional data, including conditions (low milk supply, slow weight gain, tongue-tie, latch problem, breast pain, and nipple pain), baby weights, mother’s age, birth type, preterm birth, and obstetrics/gynecology history. The mother-facing app allowed for calculating a self-assessment LATCH score [40], breastfeeding confidence score (adapted from the Mother Infant Breastfeeding Satisfaction Scale subscale of the Hill & Humenick lactation scale [41]), daily diaper counts, and a pain scale scored from 0 to 5. The questionnaire format was not independently validated.

Data were stored in separate secure cloud-based data storage servers for each app (AWS and Google Cloud). The system was established for investigational use only was not intended to diagnose or treat any medical condition nor to serve as a substitute for medical advice or guide medical care. Data confidentiality was maintained according to user consent and usage terms (see Multimedia Appendix 1).

### Data Collection

This study utilized a data set collected from July 2018 to October 2020 by lactation support providers (n=30), including International Board-Certified Lactation Consultants, that used the system at routine home visits, as well as mothers joining in their third trimester who voluntarily used the system for repeated self-assessment starting soon after birth. An additional data set used for milk sample analysis in our laboratory from 2015 to 2023 was also included for system performance assessment (for details see Multimedia Appendix 2). Participation and use were optional, and the informed consent form explained system use, system limitations, and data handling (see Multimedia Appendix 1).

For analysis, data were anonymized, manually organized, and combined from the two app data storage servers. Specific records missing vital classification data or flagged as system failures or misuse were excluded. The unstructured data collection schedule, optional data tags, data merging from two participant groups, and reliance on self-reported data present study limitations. To offer comprehensive group-level analysis, all scans were treated as independent, including multiple-day scans from the same mother, presenting a possible bias toward mothers who scanned more frequently.

### Data Analysis

We defined exclusive, full, and partial breastfeeding as previously described [42].

For data set analysis, data points were retrospectively categorized into three types of breastfeeding—“normal,” “low supply,” and “breastfeeding problems”—based on system interactions by the lactation support provider or mother. These categories reflected reported breastfeeding exclusivity and problem severity.

The “normal” classification included either exclusive breastfeeding, full breastfeeding, full mother’s own milk (as described by Labbok and Starling [42]), or predominant mother’s milk (≥80% of daily feeds, also referred to as high partial mother’s milk feeding) at the time of the scan, with no indicated problems associated with low milk supply.

The “low supply” classification included lactation support provider’s reports of formula feeding (defined as nonexclusive breastfeeding since birth and formula feeding during the 24 hours prior to the scan; also referred to as low or medium partial feeding) that were also tagged with problems associated with ineffective breastfeeding and/or low milk supply, or mothers’ reports of indirect indicators of low milk supply and slow baby weight gain or significant formula feeds (over 20% of daily feeds and up to mainly formula as the nutrition source of the baby at the time of the report). Importantly, milk supply was not directly evaluated.

The “breastfeeding problems” classification included nonexclusive, partial breastfeeding (any formula at 34 hours prior to the scan) but with no obvious tagging of problems, or predominant breastfeeding (≥80% mother’s milk at scan) also tagged with problems associated with ineffective breastfeeding and/or low milk supply (latch problems, 32.9%; tongue/lip-tie, 23.9%; low weight gain, 18.7%; and/or low milk supply, 5.2%).

Group names do not imply either a diagnosis or a confirmed clinical condition. This study is limited by the fact that classification was based on subjective user-reported suspected status, with no direct measurements of milk volumes or per-feed milk transfer by the authors. For ease of reporting, throughout this article, the short descriptors are used as noted above.

### Statistical Analysis

Statistical analysis was conducted using JASP Graphical Statistical Software Version 0.17.1.0 (JASP Team, University of Amsterdam, Amsterdam, the Netherlands). Descriptive statistics included mean, median, standard error, coefficient of variation, minimum/maximum percentiles, and quartiles. A two-factor ANOVA was used to estimate the mean change in MM% based on feeding and days after birth, followed by a Tukey test for multiple comparisons. Scatter and interval plots are used to represent numeric variables and 95% CIs, respectively. For percentile analysis, a ±24-hour smoothing window was applied for the first 3 days and a –24-hour window was applied thereafter. Data set distributions were visualized with raincloud plots and quantile limits were visualized with boxplots.

## Results

For Visual Abstract see Multimedia Appendix 3.

### Milk Sensing Device and App

The milk electric conductivity sensing apparatus used to collect data presented in this manuscript was initially tested for laboratory performance in a series of breast milk samples. Results obtained with the apparatus were found to strongly correlate with milk sodium (Na^+^), a well-studied electrolyte with regard to lactation state [14,15,19,22,23] (see Figure S1 in Multimedia Appendix 2). In our records, both the apparatus results and Na^+^ showed a strong day-after-birth–dependent dynamic change, previously linked to secretory activation [15,38], and both presented a similar association with inflammation-associated breast pain, as previously reported [29,30] (Figure S2 in Multimedia Appendix 2). Although in the scope of this study not all possible contributors can be ruled out, no such correlation was found to a set of milk components with large interindividual variability in mature milk (Figure S3 Multimedia Appendix 2).

**Figure 1:**
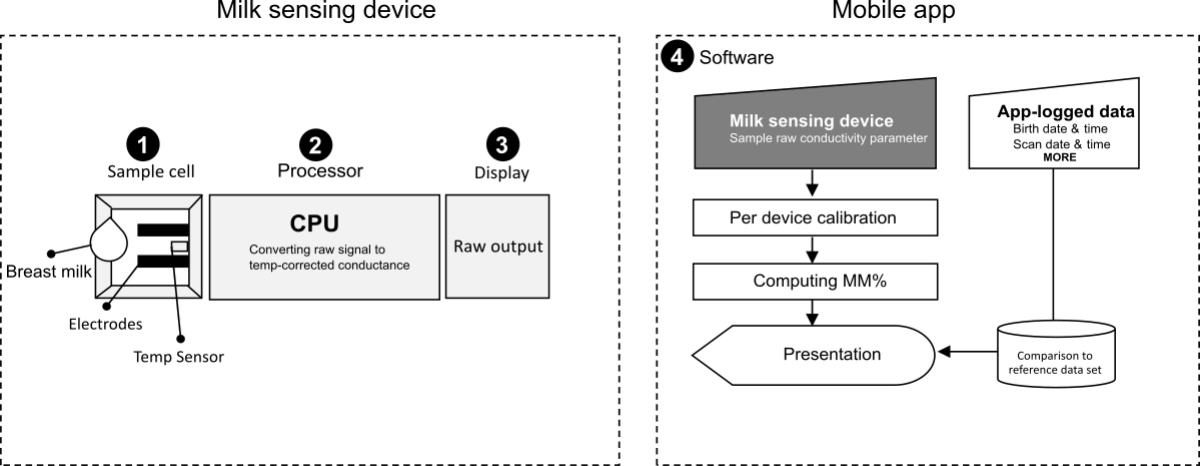
Schematic illustration of the milk sensing system. The system is composed of a milk sensing device and smartphone app. The milk sensing device design consists of (1) a sample cell with two electrodes and temperature (temp) sensor, (2) a firmware processor converting raw measurements to temp-corrected conductance, and (3) a display output. On the smartphone app side, (4) a software applies a per-device calibration on the measurable conductivity and computes the milk maturation percent (MM%) parameter. Computation is based on an empirical breast milk history data set.

The system (Figure 1) was designed to reliably sense conductivity in a small (200 μl) human milk sample (see Figure S4 in Multimedia Appendix 2) and instantly computes MM% by a proprietary equation defined based on an empirical data set of breast milk samples (detailed in the Methods section). MM% was designed to intuitively follow the directional progress of mother’s milk on a continuous 0% to 100% scale determined empirically. The system was operated by the user via a mobile app for data recording and presentation. The lactation support provider app was designed as a dashboard to manage records of different mothers, while the mother-facing app was designed to follow individual mothers’ day-to-day milk maturation progress and allow for additional voluntary data recording (see Figure S5 in Multimedia Appendix 2).

### Participants

A group of 30 professional lactation support providers were voluntarily enrolled and equipped with systems for use as an informational tool in their routine face-to-face home visits. A separate group of 37 expecting mothers (third trimester) were voluntarily enrolled for self and repeated use of the system at their home, starting early after birth. The intended use of the system was informational only and was not intended for diagnosing or treating any medical condition nor to substitute any medical advice. A retrospective analysis was performed on the observational data set recorded in the system data store between July 2018 and October 2020 as part of rolling continued data gather using the system by real-world users, mostly in the home setting.

The data set included 1511 scans, including 1098 scans recorded at 593 appointments of professional lactation support collected from 555 mothers and 421 scans recorded on the mother-facing app from mothers that used the system for repeated self-home-tracking. At the stage of analysis, the lactation support providers–derived data set included 555 mothers, with an average maternal age of 30 (SD 5.7) years, reported infant sex of 278 girls and 276 boys, average birth weight of 3224 grams, and average child number 2.2 (out of 519 records, 53% 1st, 18% 2nd, 11% 3rd, and 13% 4th+ child). Infant age at the scan ranged from 0 to 1051 days with a median of 26.6 days. The self-enrolled mother group included 37 mothers; the average baby birth weight was 3103 grams, average child number 1.8, and average gestational week at birth was 37; see detailed sample demographics in Multimedia Appendix 2. As the participating population was not controlled and was subjected to selection bias, the results of the current analysis are not intended to and cannot provide insight into distributions in the general population.

### Milk Maturation Progress in Exclusive Breastfeeding

Analysis from home use of the device demonstrated that MM% in exclusive normal breastfeeding progressed steeply throughout the first days and then gradually increased to reach a plateau within 2-3 weeks postpartum.

**Figure 2:**
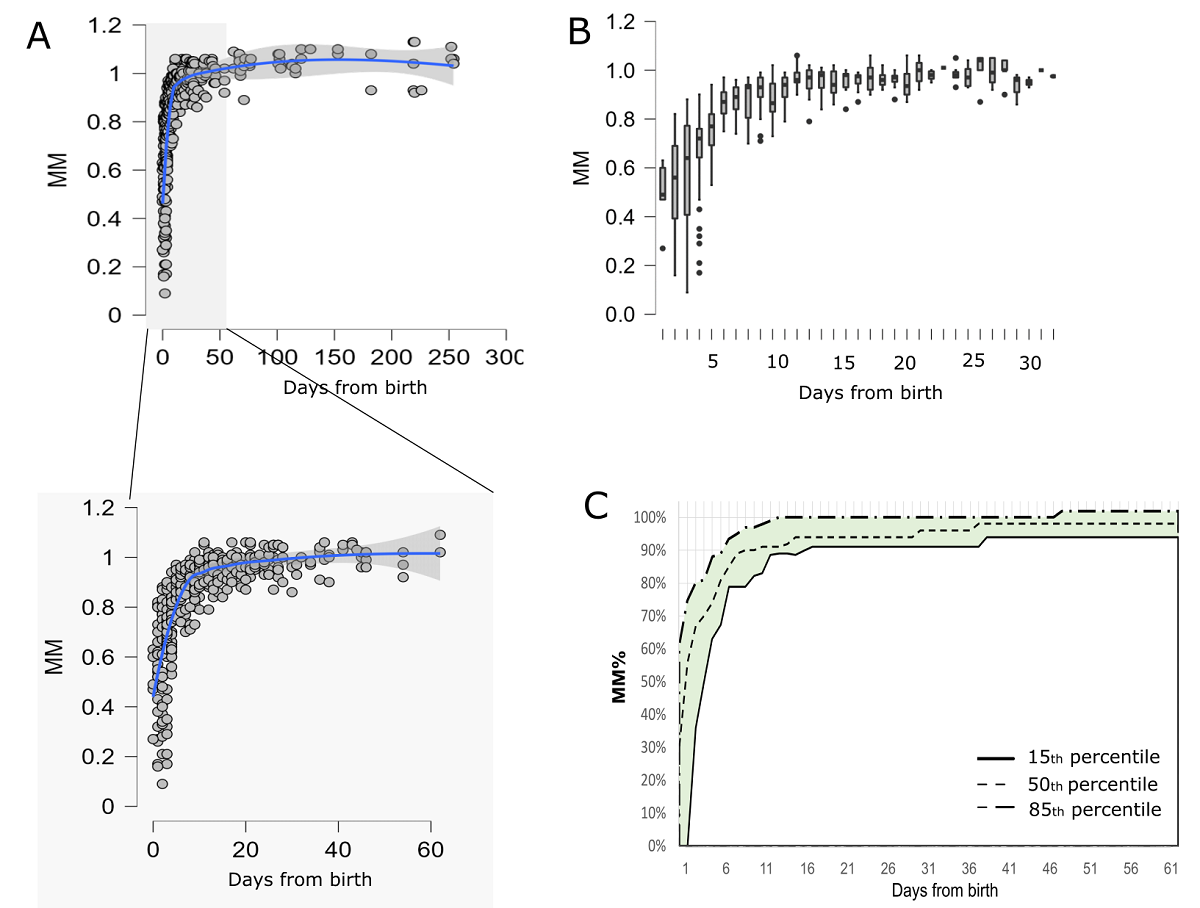
Milk maturation percent (MM%) distribution in a data set of a predominant breastfeeding population. A data set of 507 scans recorded from exclusive breastfeeding or predominant mother’s milk (≥80% daily feeds), with no problems at the time of the scan were included. (A) Dot plot chart of MM% to day from birth at scanning. The upper chart shows the full scale up to 300 days (N=507); the lower chart enlarges the 0-60 days snapshot (n=463). (B) Box plot presentation of the data distribution and quantile limits (+1.5 IQR) for each day postbirth (0-60 days). (C) MM% to day postbirth (0-60 days) chart with ranges set by the data set percentile array, where the lower full line depicts the 15th percentile, the middle dashed line depicts the 50th percentile, and the upper dotted-dashed line depicts the 85th percentile.

We analyzed a data set of scan records tagged as exclusive breastfeeding, full breastfeeding (as previously defined [42]), and predominant mother’s own milk (≥80% of daily feeds, also referred to as high partial breastfeeding) at the time of the scan, with no breastfeeding problems indicated at the time of the reporting by either the lactation support provider face-to-face evaluation or by the mother (collectively referred to as the “normal” data set). A total of 507 scans fit these criteria. Data analysis revealed rapid elevation in the MM% parameter in the first days from birth that continued to increase slowly along 2 and 3 weeks postpartum, reaching a stable plateau of approximately 100% (Figure 2A-C). High variability was noted in the first days; however, the cofactors contributing to this variation were not assessed in the current study design. We next set per-day MM% “normal” percentiles, defined at the 15th, 50th, and 85th percentiles for each day/day range (daily in the first 21 days postpartum and at 7-10–day intervals at days 21-60 and >60 postpartum) (Figure 2C). These calculations and visualizations were further used as references for single or multiple sets of measurements.

### Individual Mother’s Milk Maturation Parameter Dynamics

**Figure 3:**
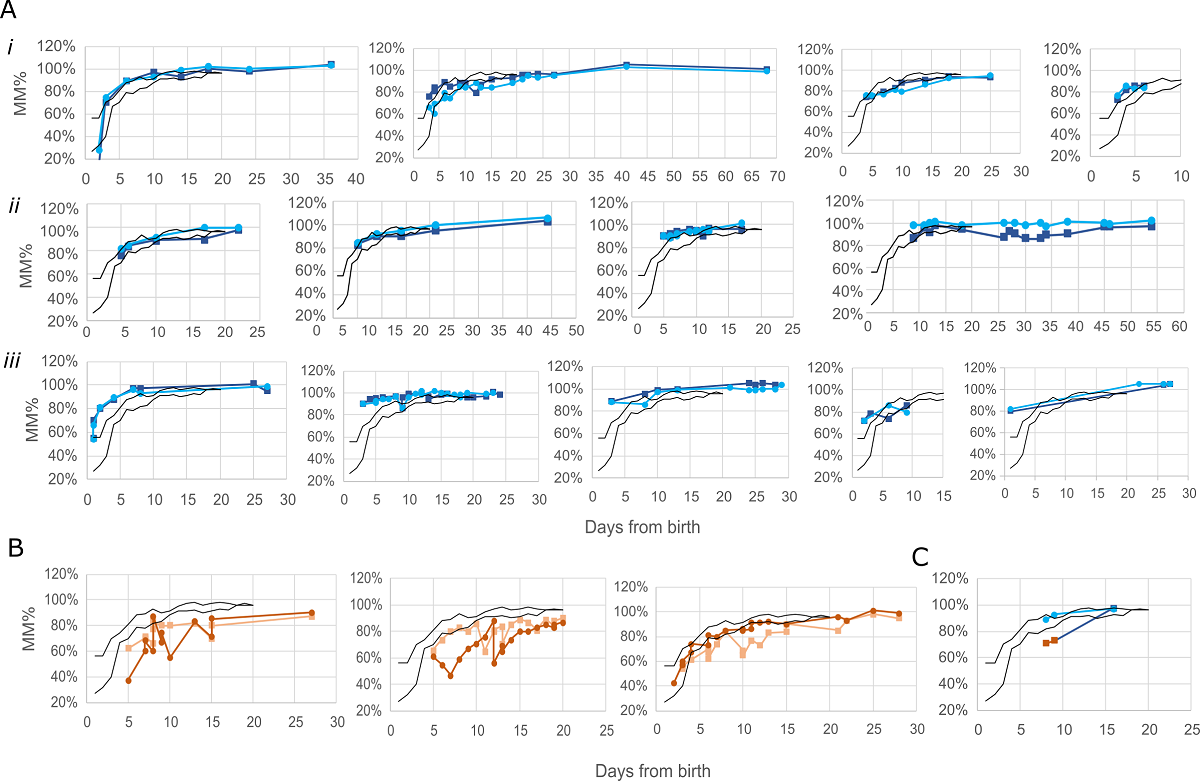
Per-case milk maturation percentage (MM%)-to-day from birth charts in individual mothers. The data set comprised 712 scans recorded by a group of 18 mothers who used the system independently at their homes. Right and left scans are depicted by separate lines. Black lines represent the 15th percentile and 50th percentile data array generated in the "exclusive" reference group. (A) MM% records from 14 exclusive breastfeeding mothers with no indications of breastfeeding problems (blue), showing similar dynamics (i, ii) or advanced (iii) relative to the 50th percentile line. (B) Three individual breastfeeding mothers with indications of breastfeeding problems of low milk supply (orange), showing slower MM%-to-day dynamics below the 15th percentile line. (C) MM% records from exclusive breastfeeding mother with delayed milk production on one side that was balanced.

Next, we assessed the dynamics of MM% in individual mothers who used the system repeatedly in a home setting (712 scans from 17 mothers that used the system on at least 4 separate occasions). The frequency of use of the device was at the mother’s sole discretion (varied between 8 and 42 scans for each mother). MM% for each breast was analyzed separately. The plot of per-mother MM% versus day (Figure 3A) revealed similar dynamics to the population medians, where MM% values increased sharply within the first days after birth and continued to change gradually within the second week, eventually reaching a plateau. Records of mothers with no apparent breastfeeding complications tended to have MM% values above the “normal” group’s 15th percentile line, often very close to the 50th percentile line (Figure 3A, i-ii). Several records presented more rapid dynamics (Figure 3A iii), reaching full (100%) maturation faster within several days from birth. In contrast, the records from selected individuals with reported breastfeeding problems associated with low milk supply reflected slower MM% versus days dynamics, where MM% values were below the 15th percentile norm line (or close to) from the early days after birth and for a period of time thereafter (Figure 3B). One record presents MM% records of exclusive breastfeeding with reported differences in milk production between breasts, with one side initially below the 15th percentile, demonstrating possible independent dynamics between sides (Figure 3C).

### Milk Maturation Dynamics in Ineffective Breastfeeding

Analysis of data from home use of the device showed that lower MM% values are associated with early breastfeeding problems indicative of a low milk supply.

**Figure 4:**
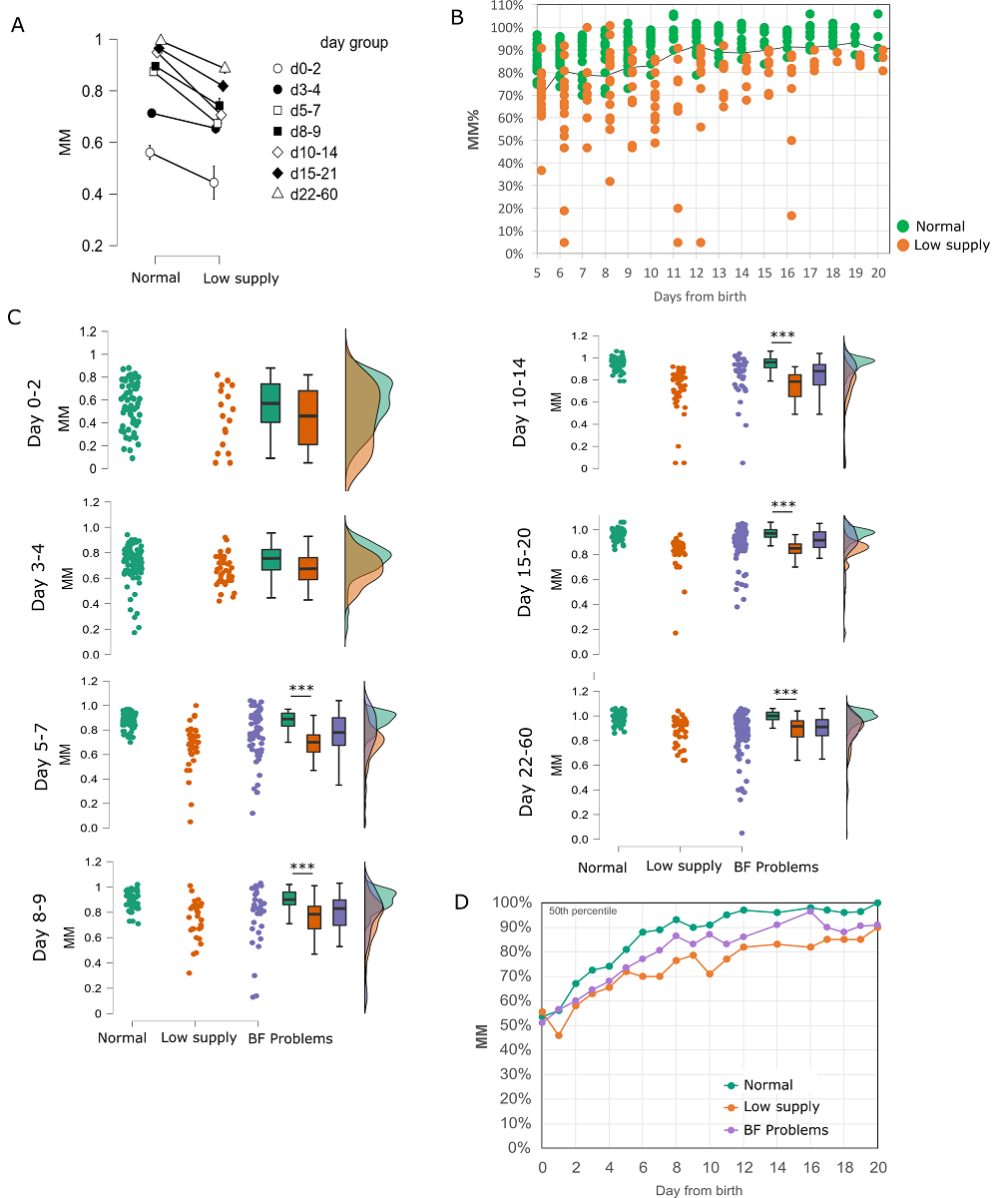
Milk maturation percentage (MM%) in population groups of low milk supply and breastfeeding problems. Analysis of scans' MM% recorded from breastfeeding women with indication of a low milk supply, as reported by face-to-face evaluation by lactation support provider or mother self-report ("low supply," n=253), compared to "normal" group of exclusive/predominant (≥80% mothers' own milk) breastfeeding ("exclusive," n=463) and a third group of diverse breastfeeding problems ("BF Problem"; n=569, purple). (A) Dot plot presentation of MM% to day from birth at day 6-20: "exclusive," green; "low," orange; the black line is the MM% 15th percentile from the norm array. (B) Per-day range comparison analysis of MM% records between the two factors "low supply" and "exclusive." Two-factor ANOVA *P*<.001, Tukey BF *P*<.001; posthoc comparisons for the interaction BF×day is statistically significant Tukey *P*<.001 at day 5 (d5) to day 20 (d20). (C) Raincloud plot visualization ("exclusive," green; "low supply," orange; "BF Problems," purple) at day ranges, showing group separation at 5-20 days from birth. Box plot presenting quantile limits (Q1, median, Q3, and ±1.5 IQR). (D) MM% group moving median per-day range at 0-20 days. Data set of days 20-60 grouped at day 20.

We aimed to test if reported early ineffective breastfeeding status and milk supply problems are associated with different MM% dynamics. The data set was retrospectively classified into one of three breastfeeding classes based on user records of breastfeeding exclusivity and reported breastfeeding problems as “normal,” “breastfeeding problems,” and “low supply,” representing the “severity” of milk supply problems (see detailed data classification and limitations in the Methods). We performed a retrospective analysis in which we compared the “normal” data set as described above to the data set of records classified as “low supply” (n=253 scan records). The low supply MM% array revealed a tendency toward lower values compared to the “normal” predominant exclusive breastfeeding classification, with lower group median, quartiles, and mean MM% for the “low supply” group compared with those of the “normal” group throughout the full period (Figure 4A-D). Per-day range analysis revealed a lower mean MM% in the “low supply”–classified records compared with that of the “normal”-classified group in every day range analyzed (Figure 4B, two-factor ANOVA *P*<.001; Tukey test for breastfeeding classification *P*<.001). Posthoc analysis showed strong statistical significance between data sets at day 5 onward after birth (days 5-20 *P*<.001; days 20-60 *P*=.002) (Figure 4C).

For the day range 6-20 after birth, 110 out of 135 records classified as “low supply” showed MM% under the 15th percentile threshold line, suggesting a potential preliminary sensitivity of 81% for using the daily 15th percentile of the “normal” predominant exclusive breastfeeding class as a potential reference threshold (see the detailed analysis in Table S1 of Multimedia Appendix 2). Future controlled prospective pilots and implementation studies, with population representative samples, direct milk supply measurements, and prolonged outcome evaluation, are required for validation of the usefulness and diagnostic capabilities of the system.

The intermediate data set, including scan records with indication of varying milder breastfeeding problems (see data classification in the Methods; n=569 scan records), showed persistent intermediate MM% median values, which were higher than those of the “low supply” group and lower than those of the “exclusive” norm values, most noticeably between days 5 and 19 (Figure 4D), with extended distribution over the full range defined by the two other groups (Figure 4C). As expected in this nonhomogeneous data set, some individual MM% records presented intermediate values in line with intermediate breastfeeding problem severity, while others were either more similar to the “exclusive” data set or to the “low supply” data set. Further metadata analysis and follow-up studies may highlight cofactors involved and their impact on the recorded MM% and possible subclassifications within and between groups.

## Discussion

### Principal Findings

Here, we present the first real-world data collected in real time using a newly developed human milk conductivity sensing system in the most intensive postnatal period and in a natural home setting. The real-world data design enabled a large, diverse, unique data set, including the MM% parameter alongside unique mother-baby data from numerous heterogeneous cases.

Retrospective analysis of the data demonstrated a typical “normal” MM% progress at the group level and in individual repeated measurements over the first days and weeks postpartum, and demonstrated that the system described is sensitive to breastfeeding exclusivity and milk supply problem severity at multiple and ongoing time frames after birth.

While randomized controlled clinical studies are warranted for evaluating the clinical usefulness compared to current practice and for validating the diagnostic capabilities of the new tool, the benefit of collecting real-time, real-world evidence in a natural setting is powerful. This can promote our understanding of breastfeeding physiology, low milk supply risks, and etiology, and serve as a step toward practical care insights.

### Building on Prior Work

Milk electrolytes are used in research as biomarkers of secretory activation, which have been highlighted as predictors of successful or failed breastfeeding [15,18,20,27]. Although precluded from routine practice, previous studies by Humenick [43], Humenick et al [23], and Morton [19] have identified the potential use of milk secretory activation biomarkers for clinicians’ per-case evaluation, suggesting that early assessment of secretory activation biomarkers can be used for early evaluation of the adequacy of lactation and the effectiveness of suckling, assist the optimal timing for a follow-up visit, enable the evaluation of progress, and evaluate the cause of inadequate lactation. A recent study validated the use of a portable instrumentation for sodium measurement directly by clinicians [44]. The system we described herewith enabled taking this theory out of the controlled setting into practice in a natural home setting, with an easy-to-operate, immediate, handheld, relatively low-cost technology, well-correlated with prior findings. The described milk conductivity sensing technology may be influenced or correlated with additional factors beyond those assessed in the scope of this study. Future controlled studies assessing various population subsets will assist in defining system usability and limitations, alongside driving further algorithm improvements.

Most studies evaluate individual data relative to a single cutoff criterion for a secretory activation biomarker level (particularly sodium). We argue that with the attempt to translate a gradually changing biomarker to a meaningful tool, an improved methodology can be to compare individual data to day-from-birth matched references. Our preliminary sensitivity assessment using per-day references in the exclusive breastfeeding “norm” population shows promise. Further studies are needed to validate norm values for a large and variable population and to evaluate or adjust the percentiles depicted in this study.

### Future Directions

Although preliminary, limited, and out of the scope of the current analysis, in a small-scale user survey, lactation support provider users verbally indicated that the system was valuable for their case assessment and follow-up (see representative quotes in Multimedia Appendix 2), raising the potential of a milk biomarker measuring system to support data-powered and remote care. The current system is built as an informational tool, not intended to diagnose or treat a medical condition and secondary to any professional care. Future studies are warranted to validate the usability and usefulness of the system and for assessing the diagnostic capabilities of the tool.

The system did not distinguish all “low supply” cases. This can be linked to primary lactation insufficiency, hypoplasia, or levels of insufficient glandular tissue [22]; cases where first use of the system was relatively late; or cases of perceived insufficient milk supply. There are certain additional conditions such as mastitis, breast inflammation, and milk stasis that are also reflected in abnormal levels of milk electrolytes [29,30]. Preliminary data suggest the ability of the system to differentiate the above cases (data not shown) and additional research is warranted for validating system use for specific intended uses.

Low milk supply is often diagnosed and managed late [9,45,46]. Insufficient lactation can reflect a variety of cases with different underlying contributing factors that can all benefit to a significant extent from as-early-as-possible preventative and corrective management. Use of the described system may assist in understanding the efforts required to increase supply. The lower the MM%, the less advanced the mother is in her milk supply establishment process, but this can also indicate that the mother’s supply can still be significantly increased with frequent and effective milk removal. Early and repeated system use can reflect day-to-day progress and may assist in evaluation of the effectiveness of management.

Using the daily milk maturation parameter and its dynamic progress throughout the postpartum period, easily measured by the mother at home with minimal effort, combined with additional data on mother and baby harbor the potential to enable (1) the classification of cases to those with lower and higher risk for a breastfeeding problem; (2) evaluating the efforts required for enhancing milk supply and to assess intervention efficiency; (3) differentiating between problems that originate in maternal physiology (glandular) to problems originating in inefficient breastfeeding (postglandular, whether it stems from the baby physiology, breastfeeding management routine, or latch); and (4) structuring a tailor-made care practice accordingly on a case-by-case basis.

A mother-facing system that will include result interpretation and enhances the milk measurement with data-tracking tools, personal insights, and educational materials, tailored to the baby age and to the milk status, may enable personal, comprehensive, empathetic, and practical guidance, and has the potential to drive a mother’s early proactivity. This was not directly evaluated in this study and should be addressed in future research, along with assessing the system’s benefits beyond the current standard of care.

### Limitations

This study was designed as a retrospective descriptive analysis of the data recorded in real-world use by early adopters assessing the technology. First, as the real-world data were not collected as part of a structured research, study limitations include potential selection bias, data inconsistency, confounding factors, and quality assessment challenges. Second, system usage was not routinely controlled and self-reported records were subjective and not complete, limiting data analysis capabilities. In an effort to minimize biases caused by these factors, we chose to include a relatively large data set in this feasibility study.

Third, the app was available only in English and only for iPhone, and the population was not randomized nor controlled but rather included a self-volunteered population that are in favor of the solution; therefore, the data set cannot and is not intended to represent the general population. Future large-scale controlled studies on a representative sample will help in addressing some of these limitations.

### Conclusions

This novel mother’s milk conductivity sensing technology used by real mothers provides a proof of concept for real-time self and remote reliable assessment of individual milk secretory activation progress in a home setting. Bringing a milk assessment technology for self and remote tracking to the hands of mothers and lactation support providers for use at home, the place where breastfeeding is normally managed in real life, and at the most sensitive and critical period of breastfeeding establishment harbor future research and care potential.

Adoption of secretory activation biomarkers sensing solutions, even as an informational-only tool, could be potentially leveraged into promoting breastfeeding rates and maternal well-being.

## Appendix

Multimedia Appendix 1 Privacy policy and consent wording.

Multimedia Appendix 2 Description of the device and laboratory testing data (Figure S1), milk biochemical correlation analysis (Figures S2-S3), sample size and stability testing (Figure S4), calculation of MM%, app screenshots (Figure S5), sample demographics, predictive analysis of MM% (Table S1), representative quotes of feedback from lactation consultant users.

Multimedia Appendix 3 Visual Abstract.

## Abbreviations

MM%: milk maturation percentage
STROBE: Strengthening the Reporting of Observational Studies in Epidemiology

## Data Availability

The data sets generated during and/or analyzed during this study are not publicly available due to data privacy and intellectual property reasons, but are available from the corresponding author on reasonable request.
