# Supplementary material for: Novel At-Home Mother’s Milk Conductivity Sensing Technology as an Identification System of Delay in Milk Secretory Activation Progress and Early Breastfeeding Problems: Feasibility Assessment
Source: JMIR Pediatr Parent. 2023 Aug 22;6:e43837. doi: 10.2196/43837 (PMC10481223; doi:10.2196/43837)
Supplement: Multimedia Appendix 1 [file pediatrics_v6i1e43837_app1.pdf]

Appendix_Privacy_policy _and_consent – relevant sections

MyMilk laboratories provides non-diagnostic breastmilk testing services.

The company is the owner of the legally registered user database (database #7000655996, Israel Privacy Protection Authority database registry.

For the milk sensing device and App use, mothers voluntarily used the system for repeated self-assessment. The system was not intended for diagnosing or treating a medical condition but rather as an informational/Educational low risk tool for promoting a healthy lifestyle. This was clearly indicated at all steps of registration. System was used in a non-clinical non-academic setting, any and all system interaction was at the sole discretion of users. System was used directly by the participants.

all users agreed to privacy policy and terms of use allowing for storing and using the data for R&D purposes, as part of registering to MyMilk services or on-boarding the milk sensing device and App use.

Specific wording:

In the App on-boarding :

Review the form below, and tap Agree if you're ready to continue.

Data privacy

When you use the mobile App, you may choose to provide MyMilk with certain optional information at your sole discretion. This information is used to enable Mylee to provide you with access to certain areas in the App, to personalize and to communicate with you about the services. We will keep your identifiable and non-identifiable information private, and will never make public any information you disclosed to Mylee during the course of using the product in any identifiable manner, unless you actively provide your specific consent. All data provided will be processed according to our full privacy policy as specified in www.mymilklab.com/milkscan_terms

Please Notice

System is Not intended for diagnosis or treatment of any medical condition, and the information provided is not a medical advice. In any concern about your breastfeeding, your baby or your own health, contact your healthcare provider.

Avoid contact of device with nipple or breast.

Do not use milk that came in contact with device parts.

KEEP AWAY FROM CHILDREN

Appendix_Privacy_policy _and_consent – relevant sections

At voluntary registration to testing community, and at LC app terms of use :

Consent form: EARLY TESTING COMMUNITY MEMBER CONSENT

THE REGISTRATION TO THE APP OR THE USE OF MYMILK SCANNER, CONSTITUTE YOUR CONSENT TO THE FOLLOWS:

YOU UNDERSTAND THAT MYLEE **DEVICE AND SOFTWARE ARE NOT INTENDED FOR DIAGNOSIS OR TREATING ANY MEDICAL CONDITION** IN THE MOTHER OR BABY, AND CAN BE CONSIDERED AS A SUPPORTIVE INFORMATION ONLY, FOR PROMOTING HEALTHY LIFESTYLE.

YOU UNDERSTAND THAT THE CONTENT PROVIDED IN THE APP **IS NOT INTENDED TO BE A SUBSTITUTE FOR PROFESSIONAL MEDICAL ADVICE.** THE COMPANY URGES YOU TO SEEK THE ADVICE OF A LACTATION CONSULTANT OR OTHER HEALTH PROVIDER IF YOU HAVE ANY QUESTIONS OR CONCERNS. SYSTEM IS SECONDARY TO PERSONAL CAREGIVER ROUTINE PRACTICE AND ADVICE. IN ANY DISCREPANCY BETWEEN THE SYSTEM AND THE CAREGIVER, FOLLOW YOUR CARE GIVER ADVICE.

You ACKNOWLEDGE THAT **THE DEVICE AND ITS USE ARE IN BETA TESTING AND SUBJECTED TO DEVELOPMENT AND CHANGES.** This means that the scanning results, and the information provided may be inaccurate or wrong, and are subject to improvements that may lead to significant changes of the result interpretation. MyMilk will not be responsible for any use of the data.

You confirm that You are willing to scan milk samples, and give MyMIlk Laboratories LTD the permission to perform the analysis. You are aware that the information as a result of the sample analysis is of a personal nature.

By using the mylee system **you choose to disclose, voluntarily, knowingly to MYMILK LABORATORIES, PERSONAL IDENTIFIERS** (such as name, e-mail, ID,) and **personal HEALTH INFORMATION** such as DATES, and other information regarding my breastfeeding practice, my baby status and my pregnancy history. You are NOT obligated to disclose any of the information, and can choose what information to include. This information is used by the system algorithm, and therefore you understand that the information provided affect the outcome. All of your non-identifiable, and identifiable information will be stored in the company's database and will be processed in accordance with the company's privacy policy.

Failure to comply with the instructions of use of the device, or providing misinformation via the App, may affect the results and their validity, and therefore the company should not be held responsible in any way for the results, or their use.

Your personal identity will be kept confidential by all those involved and will not be published in any publication. You give your explicit and irrevocable consent to **make use of the information collected at the system, in a de-identified manner (ANONYMOUSLY) and/or aggregated form** (in a manner that does not identify you or violate your right to

privacy) for the improvement of the tool, educational, marketing, and research and development purposes by MyMilk Laboratories.

All the property rights belong to MyMilk Laboratories. You understand that your participation in the pilot and/or use of the system does not entitle you any rights in the intellectual property or products developed. You are hereby confirm that you are not in contact with a company or other entity attempting to obtain information about the technologies or intellectual property of MyMilk Laboratories.

Early testing community members receive free-of-charge device and are obligated to return it after 30 days of use, starting from activation date via the App (but you can get an approval for an extended period for use). By the end of the approved period, you are being asked to recommend a friend, and pass the device to a new expecting mom, or to return the scanner to MyMilk Laboratories (Havazelet Hasharon 37, Herzeliya).

You are free to choose not to use the system at any time. You hereby declare that you have given you consent at free will and understand all of the above, and chose participate and to use the system.

Relevant sections in the full Privacy policy and terms of use :

Privacy Policy

1. **Company Privacy Policy and Disclosure**

In order to use the Site and the Services, you must first familiarize yourself with and read the Privacy Policy set out below. You may not use the Site or the Services if you do not accept the Company's Privacy Policy as it appears on this page and as updated from time to time.

2. **Collecting and receiving information**

When you use the site or the mobile Application, information about you will be collected as a user of the record or as a non-registered user, such as products and services that you have been interested in or purchased, information or advertisements read on the site, the pages viewed, the means of payment used, the location of the computer through which you accessed the site, , Internet Protocol (IP) address , the date and time of access to the site; The web address of the website from which you directly accessed the site, etc. This information is intended to help improve the site experience, to analyze the site's activity, to manage it optimally, to verify necessary information, and to protect information. Use of the Site and its services by its employees or contractors at any time and hold such information for monitoring and monitoring and statistical analysis of the Company.

Appendix_Privacy_policy _and_consent – relevant sections

The Site uses cookies for its ongoing and proper operation, including to collect statistical data about the use of the Site, to verify details, in order to adapt the Site to your personal preferences and to information security needs. “Cookies” are text files that your browser creates according to a command from the company’s computers. Some cookies will expire when you close your browser and others are stored on your computer’s hard drive. The information in the cookies is encrypted, and the Company takes precautionary measures to ensure that the Company’s computers can read and understand the information stored therein .

If you do not want to accept cookies, you can avoid this by changing the settings in your browser. To do this, please consult the browser help file. Note, however, that disabling the cookies may result in you being unable to use some of the services and features on the site or on other websites. In addition, you can delete the cookies on your computer at any time. It is suggested that you do so only if you are convinced that you do not want the site to be tailored to your preferences.

Details of the user you have provided, any additional information that may be collected about you in a manner that may personally identify you, such as your name and email, and other personal information including health information about your birth date, breastfeeding habit , baby information such as weights and your health OB/GyN submitted and the results of the milk scans if milk scanning within the Services (“Personal Information”) will be stored in the Company’s database, Are not held or retained by the Company. The Company takes a strict information security policy to maintain the confidentiality of its customers’ information in accordance with the provisions of the law. Without derogating from the foregoing, MyMilk will take all reasonable measures in the industry to ensure that your personal information is kept confidential.

Except in the event that you wish to receive services as an authorized user, you do not have to give any personal information to MyMilk and service delivery will be done according to your desire to register for the service.

If you confirm this during the registration process (by clicking or checking in the appropriate place) or the order or during the use of the Site or Application, marketing and advertising information may be sent to you from time to time by e-mail. You may cancel this at any time by contacting us on the Site. The Company will not forward your personal information to advertisers.

THIRD PARTY SOFTWARE/SERVICE PROVIDERS. We may use UXCam, which is an analytics solution. UXCam may record: Screens visited, Interaction patterns (such as screen actions, gestures: taps, scrolls) and Device details (Type, Version, Model, Operating System). We are using the information collected by UXCam to improve our app. UXCam does not collect personally identifiable information and does not track your browsing habits across apps. For more information see UXCam- Privacy Policy for Information Collected by the UXCam Service.

3. **How to use the information received and collected**

Appendix_Privacy_policy _and_consent – relevant sections

The use of personal information and other information about you will be done solely for the purposes set out below, subject to the provisions of any law:

1. To provide you with various services, including the creation of personal account where you can receive milk results, computerized recommendations, personalized recommendations and general recommendations will be displayed, as well as the purchase of products and services on the Site.
2. To improve and enrich various services and the content offered by MyMilk, in order to create new services that meet the requirements of the users of MyMIlk services and their expectations, and to modify or cancel existing services.
3. To create an anonymous database of different habits and composition of breast milk. The database will be used for analysis, control or research and development purposes. In addition, the Company may share anonymous and non-identifying information with non-profit organizations or commercial partners for research.
4. To match the ads you see when you visit the site to your interests. The information used by the Company for this purpose will not personally identify you by name, ID or address. However, this information, which does not personally identify you, may be transferred to third parties, including advertisers.
5. To contact you through the Site or by email to provide the Services, accompany at various stages of the process as part of the Services or in addition to the Services or after termination of the Services for the dissemination of additional relevant information including advertising.

4. **The right to review the information and request to change or delete**

you are entitled to request in writing that the company delete information in the Company's databases used for personal contact with you. The Company will delete the information it needs to contact you personally, as requested. Information required by the Company for the conduct of its business, such as documentation of personal and commercial activities you have performed on the Site, will continue to be maintained by the Company in accordance with law, but will no longer be used for your inquiries. In order to contact the Company, please email me to the following address: contact@mymilklab.com or to the offices of the company.

5. **Sharing information**

The Company does not publish or share the results of your tests (except in an aggregate, processed, and non-personally identifiable manner) or other personal information without expressly requesting and receiving your prior permission, unless it is required to do so by law. You agree that the Company may keep and disclose personal information to other statutory authorities and bodies if required to do so by law, court order or other authorized body by law or in good faith belief that such disclosure is reasonably required to (a) comply with the requirements of the (B) in order to enforce the Terms of Service (c) to respond to contentions that infringe the rights of third parties; (d) to enforce the Terms of Service,

Protect the rights, property and personal security of the Company, its employees, contractors, users, customers and the public; or (e) Vera, that the disclosure of information is necessary to prevent serious damage to the body or body of a third party. In this case, the Company will notify you by email or the address you provided to the Site as part of the User's information, unless such notice violates any law or court order.

This Privacy Policy is an integral part of the Terms of Use and will be read and considered as an integral part thereof.

Read the Early testing community consent form before use at https://www.mymilklab.com/testingcommunity_consent

Finally, in order to continue the research in which the company deals with and improve its tools, services offered to you and others, and other research and development purposes by MyMilk Laboratories you give your explicit and irrevocable consent to the company to make use of the information collected at the system, in a de-identified manner (ANONYMOUSLY) and/or aggregated form (in a manner that does not identify you or violate your right to privacy). You will not be notified of any specific use. You understand that sample scanning, or providing information about yourself, does not entitle you rights to research or products developed by MyMilk. And you hereby grant the company an irrevocable license to utilize the de-identified personal information for any scientific, educational, marketing, and research and development use by the company.

When used by lactation consultants, LCs were instructed not to provide any identifiable information of mothers of which samples used for scan, and MyMilk did not have any direct contact with the mothers.

At voluntary registration to MyMilk laboratory testing services :

Obligations and consent

In order to enable us to provide the Services, you must undertake and declare as follows:

1. Information learned through the site is not intended for the purposes of self-diagnosis, prevention or treatment of diseases or for health diagnosis in the absence of other medical or clinical information. You understand that while the company may diagnose or suggest treatment or possible change in the habit of nutrition, this should be verified and supplemented with medical information and individual consultation with a qualified doctor, nurse, nutritionist or nursing counselor. The company urges you to consult a doctor or other medical provider if you have any questions or concerns regarding the milk test .
2. You give the Company and anyone acting on its behalf permission to perform milk tests on the milk sample and you ask the Company to disclose this information and the results of the examination to you and the others you have approved.

3. You confirm that you are between the ages of 20-45 and that you give a sample and authorize us to analyze and test the sample in our laboratories. If you confirm these Terms of Use on behalf of another woman for which you have legal authority to perform these acts, you confirm that you have such authority and that the sample is provided by a woman who meets the conditions in these Terms of Use and that the sample is given voluntarily, knowingly and without coercion.
4. If you are outside the borders of the State of Israel, you confirm that this action is not subject to import prohibitions from the country in which you live or from which you receive the service. And you confirm that you have demonstrated that the transfer and any processing of information may be transferred or processed outside of the country in which you live or receive the service. It is hereby clarified that it may not be possible to transfer the sampling or the results in accordance with the provisions of the law or regulation that apply in any case and you agree that the Company will have no obligation in such a case.
5. You warrant that you are not a company engaged in data collection or analysis, insurance company, private detective, employer or other entity trying to obtain information about a woman through the site and services.
6. You are aware that the information you will receive as a result of the sample analysis is of a very personal and sensitive nature.
7. You take responsibility for all possible consequences of sharing your account or lab results with others, and the information you have provided to the site.
8. You understand that all of your personal information will be stored in the company's database and will be processed in accordance with the company's privacy policy and by law.
9. You understand that by supplying samples and processing the specimen or providing additional information about yourself, you have no right to research or commercial product that may be developed by the Company or that the Company will improve with the information you have provided. You will not receive any compensation or money for any research or commercial product that will include the results of the information from your test or from information you have provided yourself to the site.
10. You hereby declare that you have the authority, under the laws of the State of Israel and under the laws of the country in which you live or receive the Service, to secure and maintain such representations and warranties. In the event of a breach, the Company has the right to freeze or terminate your account and refuse any or all of your current or future use of the Services. And you will indemnify the Company against any demand, cost or damage caused to it due to a breach of the representations and warranties above.

Finally, in order to continue the research in which the company deals with and improve its services offered to you and others you give your explicit and irrevocable consent to the company to make use of the sample residues, if any after completion of the test and the analysis for which the sample was given, for research and development of the

company. The Company shall be entitled to make full use of the sampling remains in its sole discretion and for its research and / or commercial purposes, and there shall be no claim regarding such use provided that the Company's use of the specimen will be in a manner that does not identify you or infringe your privacy in accordance with the Company's privacy policy, according to any law.
