# Supplementary material for: Novel At-Home Mother’s Milk Conductivity Sensing Technology as an Identification System of Delay in Milk Secretory Activation Progress and Early Breastfeeding Problems: Feasibility Assessment
Source: JMIR Pediatr Parent. 2023 Aug 22;6:e43837. doi: 10.2196/43837 (PMC10481223; doi:10.2196/43837)
Supplement: Multimedia Appendix 2 [file pediatrics_v6i1e43837_app2.pdf]

SUPPLEMENTARY MATERIALS

Device apparatuses laboratory testing

Devices (proprietary, in-house assembled apparatus, MyMilk Laboratories LTD) were tested with KCl standard solutions to determine linearity in the measuring range of 3-50mM (471-6654 µS/cm; representative calibration curve presented in **Supplementary Figure S1A**, R^2=0.995). Linearity performance in the expected measuring range were set as an acceptance criterion for the individual device performance.

Two assembled devices were tested for laboratory performance testing including precision, accuracy and repeatability. Prior to performance testing, the devices were checked by series of 7 concentrations of KCl 3-50mM (471-6654 µS/cm) by 8-10 runs in different days (Averaged %CV; Device 1 %CV=5.5%; Device 2 %CV=5.8%), and the averages were used to set each device individual calibration.

Accuracy performance was tested with 5 concentrations of KCl solutions within the range of 3-40mM (471-6654 µS/cm), repeatedly tested in 5 different days, and recovery was calculated based on the error rate relative to the expected values (Device 1: Recovery=94% (%CV =5.7%); Device 2: Recovery=92% (%CV= 0.8%)).

Device Precision was tested by repeated measuring of 4 level KCl solutions within the measuring range of 3-30mM (471-4023 µS/cm), in 5-6 different days. Coefficient of variation was calculated for each device (Device 1: 3 concentrations, 6 repeats, %CV= 4.1%; Device 2: 4 concentrations, 5 repeats, %CV=3.6%).

Device precision performance was further tested in a series of frozen breast milk specimen. Milk samples were selected from MyMilk stored sample set, derived from samples voluntarily sent by mothers for various informational lab tests. Mothers provided voluntary additional data and provided a waiver and a consent for further anonymous use of remaining samples for internal R&D by the company. Milk samples were kept frozen (-20c) in 1 ml aliquots. Before analysis, either by laboratory grade instruments or the evaluated apparatus, samples were brought to stable room temp. Eleven samples were used for repeated measurements, 4 different breast milk samples and 7 spiked milk samples with elevated concentration of KCl to cover the wide range of concentrations that reflect milk samples from various time points and states (sample average conductivity range 2193-7614 µS/cm). Breast milk Samples were tested by the devices in 5-9 different days to evaluate precision. Coefficient of variation was calculated for each set

of testing (Device 1: non spiked samples, n=4, 5 days repeats, %CV=3.5%; Spiked samples, n=7, 9 days repeats, %CV=6.8%; Device 2: spiked samples, n=7, 5 days repeats, %CV=10%).

Device performance and stability for long periods were tested by evaluating system repeatability within a period of several months. The device was monitored for 11 months by repeated testing of 5 levels of KCl solutions (3-50mM, 471-6654 µS/cm)), and for 8 months period with repeated scanning of 5 frozen breastmilk samples (2598-6483µS/cm). CV% was calculated from tests on 6-8 separate dates distributed along the assessment period (KCl solutions, n=5 samples, 8 repeats, CV=4.7%; Milk samples, n=5 samples, 6 repeats, CV%=3.8).

We further compared the results obtained by device apparatus to results obtained for a serial of large number of breast milk samples by lab-grade conductivity meter (LAQUA Twin conductivity meter EC-33, HORIBA, (**Supplementary Figure S1B**, full range 1475-5260 uS/cm, n=79, R^2=0.95, Recovery=95.8% (CV% 6.3)).

To understand the relation between the apparatus measurement and sample sodium concentrations, to this large set of milk samples, we plotted the measured relative conductivity against the sodium concentration measured for each sample. Milk sodium was measured in de-fatted breast milk by laboratory grade ion selective electrode module (The ISE module of the Roche/Hitachi Cobas 6000 c501 system), and in whole milk sample by portable Na^+ ion selective electrode analyzer (LAQUA Twin, NA-11 HORIBA), apparatus that has been reported to be validated for accurate analysis of human milk sodium levels [37]. Results were found to be well correlated supporting Na as a major contributing factor to Milk conductivity (**Supplementary Figure S1C**, measured milk sodium full range 4-53 mmol/L, n=79, R^2=0.82, 0.86 respectively).

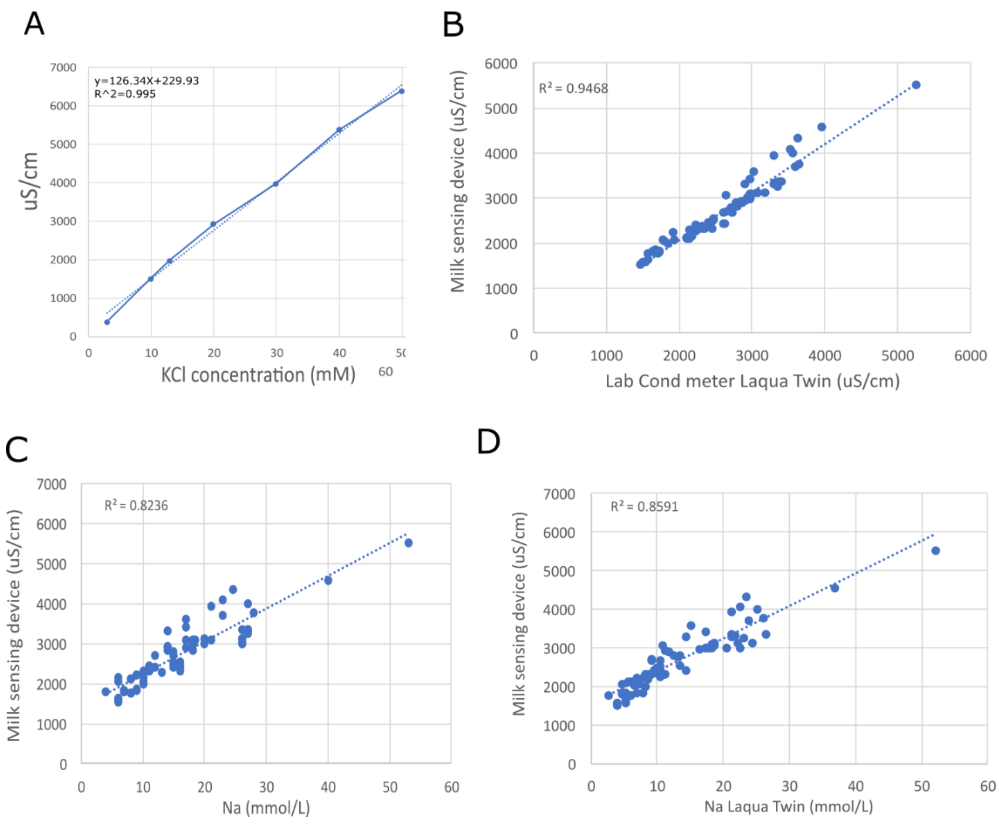

Supplementary Figure S1. Laboratory testing of the device apparatus performance. **A.** Representative calibration curve with KCL standard solution in the measuring range of 3-50mM (471-6654 µS/cm), presenting result measurements in uS/cm, calibration equation and R^2. **B-D** Dot plot chart presenting the correlation between the results of a series of 79 breast milk samples, measured by the handheld apparatus against the lab-grade conductivity meter (**B**, LAQUA Twin conductivity meter, HORIBA), against the sodium concentration measured in the de-fatted sample of the same milk by lab grade ion selective electrode module (B, Roche/Hitachi Cobas system), or against whole milk sample tested for sodium by a portable ion selective device (**D** LAQUA Twin ion sodium electrode, HORIBA)

Milk biochemical correlation analysis

For testing the correlation between milk conductivity and various milk components and conditions, we performed a retrospective analysis of a dataset extracted from MyMilk's laboratory database. Records were generated empirically by in-house laboratory-based breast milk testing. MyMilk laboratories provides non-diagnostic breastmilk testing services, and routine Breast milk samples are received for testing for nutritional composition analysis, breast pain origin assessment and R&D purposes. Dataset include voluntarily reported information about birth date, habits, breastfeeding status and additional maternal and baby indicators. The

company is the owner of the legally registered user database (database #7000655996, Israel Privacy Protection Authority database registry), and all users agreed to privacy policy and terms of use allowing for storing and using the data for R&D purposes. Data was retrospectively extracted depending on the analyte required for analysis.

Breastmilk testing procedures include high pressure liquid chromatography (HPLC WATERS Alliance e2696, Photodiode Array detector W/ICS 2998, Fluorescence Detector 2475, Empower 3 software), for Vitamins A (total retinol), Vitamin B1 (Th+TMP), Vitamin B2 (Rb+FAD), Vitamin B6 (PL), caffeine (+Paraxanthine), electrochemiluminescence immunoassay for vitamin B12 (in-house method based on Cobas 6000 e601 system, E170), colorimetric assay for protein (TP2, Cobas 6000, c501 system) ultrasonic analyzer for Fat (Custom adapted Julie HMA, scope electric). Samples are routinely tested for conductivity and electrolytes.

For comparing the dynamics of milk conductivity data to milk sodium (Na^+), a known secretory activation marker, we first plotted the milk parameters relative to days from birth, revealing similar sharp decline in the early postpartum period (**Supplementary Figure S2A,B**), similar to the reported dynamics of milk Na^+ in the lactogenesis phase [15]. According to previously reported link between milk Na^+ level and breast inflammation [29], we analyzed a second laboratory-generated dataset generated from samples reported and tagged with inflammation associated breast pain, and observed elevated milk Na^+ and milk conductivity in the inflammation associated breast pain dataset, compared with the normal dataset (**Supplementary Figure S2 A,B**). High correlation was found between milk sodium levels and milk conductivity levels in this dataset as well (Pearson's r=0.918, *P*<.001 **Supplementary Figure S2 C**). Analysis of cases with inflammation-associated breast pain beyond day 5 postpartum (N= 50 normal, N=19 Pain), show significantly higher milk conductivity (2487±83 µS/cm vs 3774±151 µS/cm, ANOVA F(67,1)=61.74 *P*<.001) and higher Na^+ in pain classified group (8 vs 13 mmol/L, , ANOVA F(67,1)=6.84 *P*=.01) **Supplementary Figure S2D**).

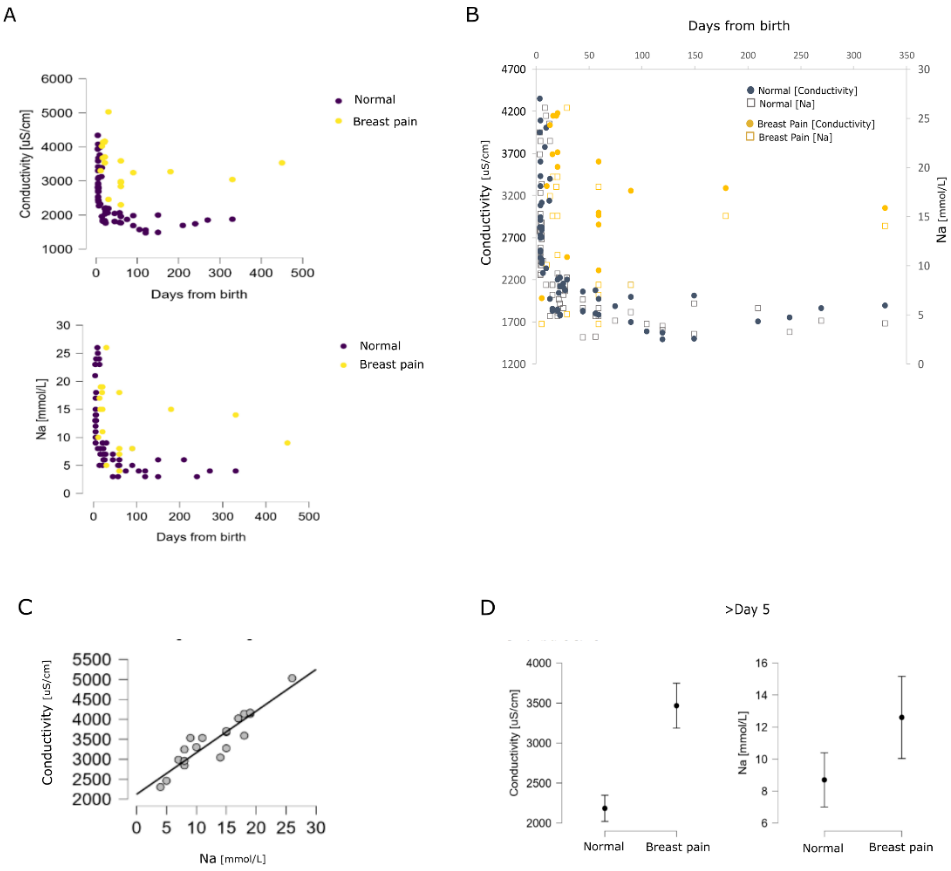

Supplementary Figure S2. Data analysis of milk conductivity and milk sodium (Na^+) in a laboratory-based datasets of reported inflammation-associated breast pain (breast pain) relative to normal dataset. **A.** Scatter plots of milk conductivity (upper panel) or milk Na^+ (lower panel) relative to day post birth (x-axis), in normal (n=65, dark) and breast pain (n=19, yellow) datasets. B. Scatter plot of milk parameter over days from birth, overlay milk conductivity (Left y-axis; circle) and milk Sodium (secondary Right y-axis; square), of normal (Dark) and breast pain (yellow) dataset. **C.** Pearson's correlation analysis between milk Na^+ and milk conductivity in the breast pain dataset (r=0.918) *P*<.001 **D.** Interval plots of milk conductivity (left panel) or milk Na^+ in normal and breast pain dataset beyond day 5.

We further performed correlation analysis to a set of milk nutrients including breastmilk main macronutrient Fat (and derived Energy), several Vitamins that are known to be effected by diet and health (Vitamins A, Vitamin B1 (Th+TMP), Vitamin B2 (Rb+FAD), Vitamin B6 (PL), Vitamin B12), and Caffeine (including paraxanthine) - a recreational drug with suggested implications on body fluid-electrolyte balance [39] , levels of which in milk are directly influenced by intake. All

these nutrients are known to have large inter-individual variability in mature milk of different mothers based on diet, lifestyle choices and health conditions.

While analysis demonstrated strong correlation between Conductivity and Na^+ (Pearson's r=0.931, *P*<.001), Fat, Vitamins and Caffeine correlations with either Na^+ or Conductivity was weak (r<0.4) to very weak (r<0.2) (Pearson's correlation analysis r<0.4, **Supplementary Figure S3A,C**).

While milk Protein levels are known to show little inter-individual differences in mature milk, it was previously shown[4], among other components, to have similar dynamics to milk Na^+ in the lactogenesis process. In Our analysis, Protein indeed demonstrated strong correlation with both conductivity (Pearson's r=0.817, *P*<.001) and Na^+ (Pearson's r=0.931, *P*<.001). Conductivity, Na^+ and Protein demonstrated similar sharp day-dependent dynamics in the early postpartum period (**Supplementary Figure S3 A,B**).

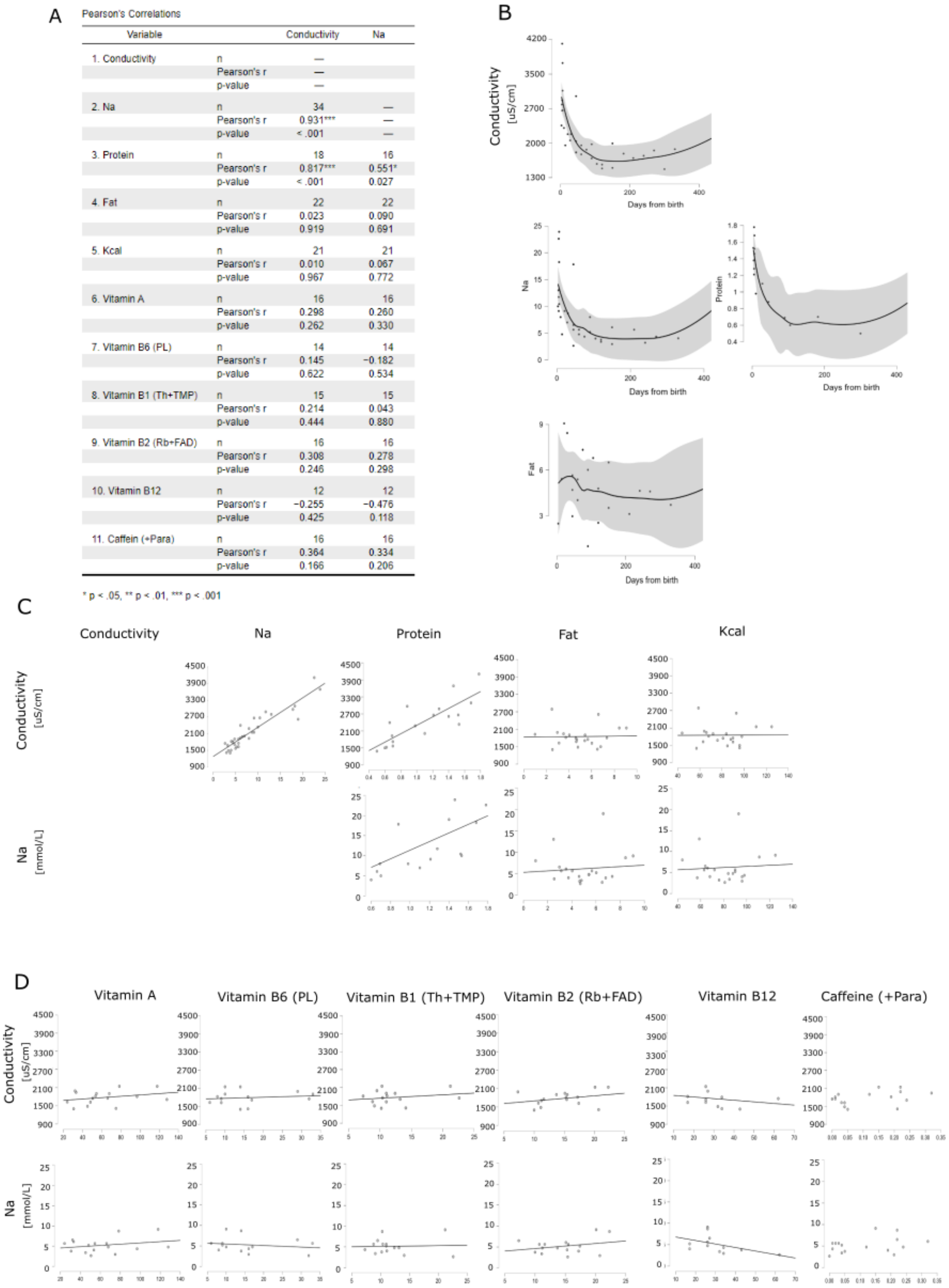

Pearson's Correlations

Variable		Conductivity	Na
1. Conductivity	n	—	
	Pearson's r	—	
	p-value	—	
2. Na	n	34	—
	Pearson's r	0.931***	—
	p-value	< .001	—
3. Protein	n	18	16
	Pearson's r	0.817***	0.551*
	p-value	< .001	0.027
4. Fat	n	22	22
	Pearson's r	0.023	0.090
	p-value	0.919	0.691
5. Kcal	n	21	21
	Pearson's r	0.010	0.067
	p-value	0.967	0.772
6. Vitamin A	n	16	16
	Pearson's r	0.298	0.260
	p-value	0.262	0.330
7. Vitamin B6 (PL)	n	14	14
	Pearson's r	0.145	−0.182
	p-value	0.622	0.534
8. Vitamin B1 (Th+TMP)	n	15	15
	Pearson's r	0.214	0.043
	p-value	0.444	0.880
9. Vitamin B2 (Rb+FAD)	n	16	16
	Pearson's r	0.308	0.278
	p-value	0.246	0.298
10. Vitamin B12	n	12	12
	Pearson's r	−0.255	−0.476
	p-value	0.425	0.118
11. Caffein (+Para)	n	16	16
	Pearson's r	0.364	0.334
	p-value	0.166	0.206

* p < .05, ** p < .01, *** p < .001

Supplementary Figure S3. Retrospective correlation analysis of laboratory-based Dataset of breastmilk components. **A.** Pearson correlation analysis for milk conductivity and sodium, with a set of milk components (Protein, Fat, calculated Energy (Kcal), Vitamin A, Vitamin B1 (Th+TMP), Vitamin B2 (Rb+FAD), Vitamin B6 (PL), Vitamin B12, and caffeine (+Paraxanthine). Number of samples for each comparison is reported as n. **B.** Days from birth distribution plots of milk conductivity, sodium, protein and Fat. **C, D.** Correlation plots of either milk conductivity or milk Na^+ with other tested milk components (**C.** Protein, Fat, Kcal; **D.** Vitamins A, Vitamin B1 (Th+TMP), Vitamin B2 (Rb+FAD), Vitamin B6 (PL), Vitamin B12, and caffeine (+Paraxanthine). Th = Thiamine; TMP = Thymidine Monophosphate; Rb = Riboflavin ; FAD = flavin adenine dinucleotide; PL = Pyridoxal.

Sample size and stability testing

Sample cell configuration was tested for minimizing the sample size. Sample adaptors, designed to hold 0.2ml, 0.5ml, and 1ml around the electrodes were tested using a set of seven KCl solutions (3-40mM). Conductivity in uS/cm were compared following calibration correction. No differences were identified between the different sample volumes in 3-20mM KCl standard solutions, with small difference in the 30-40mM that are not physiologically significant (**Supplementary Figure S4**). There was also no consistent trend of change direction with reducing sample sizes. Results were also verified with a set of frozen-thawed breastmilk samples (1220-5300µS/cm) in series of experiments (Data not shown).

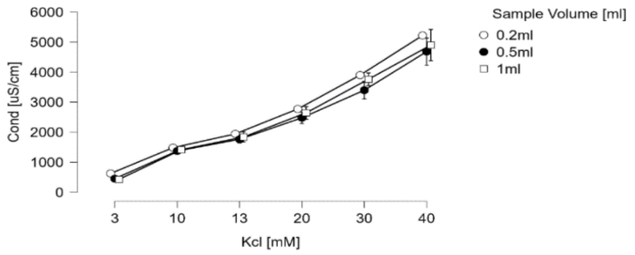

Supplementary Figure S4. Laboratory testing of sample volume cell configurations on device apparatus performance. Descriptive Plot of milk conductivity in uS/cm following calibration correction (y-axis), as measured by three types of sample volume cell configurations (0.2ml white circle; 0.5ml black circle; 1ml white square) in set of KCL standard solutions in the measuring full range of 3-40mM (x-axis).

System intended use is with fresh milk samples, however, for defining system limitations, we performed milk sample stability testing at room temperature (N=6 ; 5 time points) and following a freeze-thaw cycle to evaluate degradation effects on system's conductivity sensing. Results are stable for 4hr at room temperature (%CV 1.3±0.3%). At 22-44h in room temp, a small but non physiologically significant differences noted (average %error of 9±1.5%). This was also notable following freeze thaw cycle (average %error of 7.3±3.3%).

Setting MM% equation

MM% is computed based on the equation MM%= 1-[(X-Xmin)/(Xmax-Xmin)]*100, where X is the raw device output, and Xmin and Xmax are the pre-set. To defined the pre-set limits, min value (Xmin) and max value (Xmax), an empirical dataset of 625 scans were used (Baby age at scan: day 0 - 10 days, heterogenic breastfeeding status (exclusive/some/significant formula, with/wo problem) were used to reflect the full range; n=625; Full range 1095-9814µS/cm, Mean±STD 3761±1177µS/cm). Dataset showed A Gaussian bell-shape probability histogram with acceptable asymmetry probability distribution of a random variable about its mean (Skewness 1.32) and no heavy tailed (Kurtosis 2.88). Dataset 2.5th and 97.5th percentiles were set as Xmin and X max, respectively, for MM% equation.

Apparatus usage for milk sensing. User guide included Instructions for milk collection, and device use directly by the LC / mother. Users were instructed to collect 0.3-0.5ml milk sample, from each breast separately, either before a breastfeeding session or >1 hour from the last feed, and to scan as soon as possible to sample expression. Adequate sample volume is visible by milk appearance in a control slit in the milk cell. The value appearing on the device LCD screen after 5-10 seconds is recorded into an App (Mothers App: MyLee, iOS, ID1533231342, MyMilk laboratories LTD ; Lactation support provider App: MyMilk scan by MyMilk Laboratories (invitation only), built with Appsheet) (**Supplementary Figure S5**). Users were instructed to reject a read due to technical error If the number was not stabilized within several seconds, and to repeat the scan. Users were instructed to wash the device between scans and at the end of use, by rinsing the milk chamber tip under running tap water and air drying. The use of the device was on the sole discretion of the user, and was not monitored for compliance.

Common technical problems included inaccurate device performance due to disconnected or leaky sample chamber, battery corrosion or low voltage, dirty chamber or electrodes, or mis-entry of result to App, and users were encouraged to periodically monitor QA by provided KCl solutions. Common incorrect usage by users were mainly linked to water remains in the chamber, partial fill up due to small milk volume mainly in first usages or non-complete transition of the sample into the sample cell. Users were guided on good practices and regarding common technical issues, and were supported remotely with any technical problems.

A LC-facing App

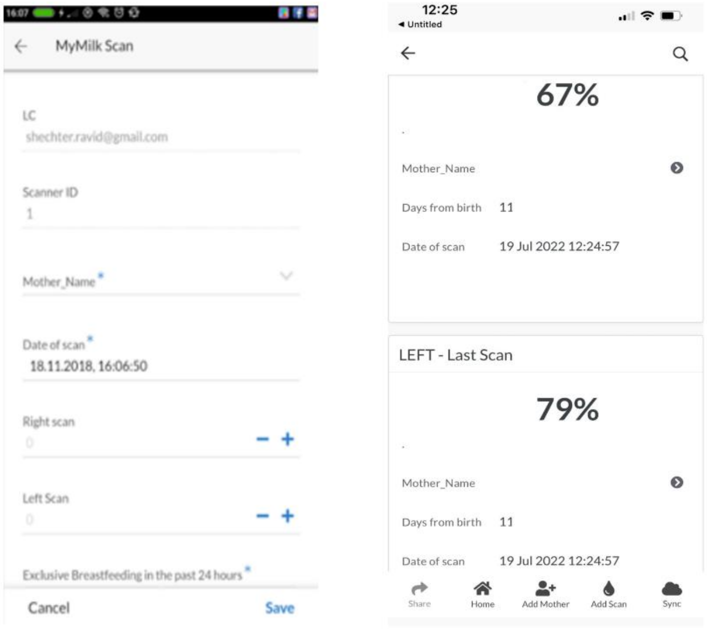

B Mother-facing App

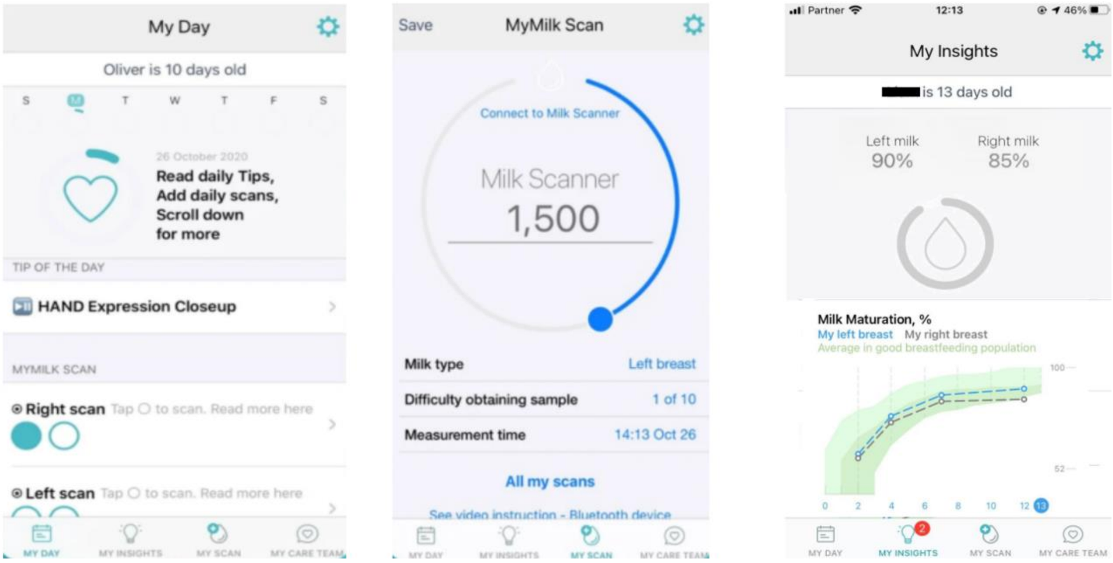

Supplementary Figure S5. Representative screens from LC-facing App (A) and Mother-facing App (B), for milk scan recording and MM% presentation.

Sample demographics Analysis

At the stage of analysis lactation support providers derived dataset included 555 mothers, with average maternal age of 30 (SD 5.7), with equal distribution between baby gender (278 Girls, 276 Boys), with average birth weight of 3224g and child number 2.2 (out of 519 records, 53%

1st; 18% 2nd; 11% 3rd; 13% +4th). Infant age at scan ranged 0-1051 days, with enrichment in the first weeks and median of 26.6 days. According to LC provided information, at on-boarding 40% of registered babies were fed only mother milk since birth, 25.4% were predominantly fed breastmilk (over 80% of daily feeds), 27.5% were fed significant or mainly formula at on-boarding (7% data was not tagged). Further Maternal and infant demographics and data were non mandatory to report. 55% of records included additional data index regarding Latch problem (20.3%), low milk supply (13.6%), Tongue/Lip-tie (23.7%), Near term (5.2%), preterm (3.6%), infant jaundice (12.9%), C-section (9.5%), and low weight gain (11.5%). Self-enrolled mother group included 37 mothers, 95% of them reported to be predominant breastfeeding/pumping at first scan (mothers' own milk over 80% of baby feeds) and 40% exclusive breastfeeding. Average baby birth weight was 3103 grams, average child number 1.8, and average gestational week at birth was 37.

Preliminarily Analysis for the predictive capabilities of the MM%

We aim to tested the predictive capability of the MM% for identification of 'Low Supply' cases. Previous research suggested a specific cutoff, for sodium levels at day 3 or day 7 after birth [19, 23], and more recently Murase et al. [22] suggested an unbiased approach for cutoff based on an analytic subset of the dataset (>75th percentile, within the same group). As milk biomarkers undergo a dynamic change in the secretory activation process, we set to apply a per-day or per-day-range MM% reference cut-off array (MM% thresholds for various day-range after birth), defined empirically in breast milk from Normal exclusive breastfeeding. We thus tested the preliminary predictive capability of the MM% at the 15th percentile of the 'exclusive' group to serve as a threshold for identifying 'low supply' cases at days 5-20 after birth. Preliminary sensitivity, and positive predictive value (PPV) were assessed versus mother's and/or lactation consultant reports of breastfeeding difficulties associated with low milk supply. Sensitivity meaning how many of those milk associated difficulties appear below threshold, whereas PPV reflects the rate of those cases below threshold truly identified with low supply associated difficulties. Preliminary analysis revealed sensitivity of 81% (110 true positive cases identified using the 15th centile threshold, out of 135 low supply cases in total), and PPV of 79% (110 true positive cases identified out of 139 cases identified in total) for day range 6-20. A day specific analysis revealed increased predictive capabilities between days 10-15 (Sensitivity=91%; PPV=88%; full day specific table provided in **Supplementary Table S1**). Analysis of earlier days (day1,2,3,4,5) or of later days (day 21-28) resulted in somewhat lower sensitivity (data not

presented). Though indicative of the potential predictive power of the test, this preliminary analysis is limited, and subjected to dataset and experimental design drawbacks. and stressing the fact that there are no gold standard lab tests for low milk supply to compare to. Failing of the test to clearly separate low cases from norm at day0-4 and beyond day20 may be linked to the tested dataset sample size or its standard deviation, but stresses the importance of repeated testing throughout breastfeeding establishment period and adding additional factors to the analysis.

Day from birth	True Positive	Positive True class (TP+FN)	%Sensetivity TP/(TP+FN)
6	12	15	0.80
7	6	9	0.67
8	8	17	0.47
9	10	13	0.77
10	9	12	0.75
11	7	8	0.88
12	11	11	1.00
13	8	9	0.89
14	6	6	1.00
15	10	12	0.83
16	7	7	1.00
17-20	16	16	1.00
Total (d6-20)	110	135	0.81

Supplementary Table S1. Preliminary predictive analysis of the MM% set by the 15th percentile array of the norm 'Exclusive' group to serve as a day-match threshold for positive identify cases of 'Low', within the period of day 6-20 after birth. Sensitivity was calculated versus mother's and/ lactation consultant reports of breastfeeding difficulties associated with low milk supply (N=135).

Lactation consultant active users verbal feedback

12 lactation consultants, who actively used the system (at least 3 separate cases) completed a feedback survey about their experience using the system. All 12 reported that the system was beneficial (9 highly useful) to their practice. User were asked to describe, in their own words, what they liked, disliked or suggest to improve in the system. selected feedback regarding use-cases are presented herewith (*Translated from Hebrew):

- “It confirms my decisions, and helps to get feedback on the amount of efforts needed to increase milk supply”
- “It helps to objectively monitor milk coming in” “Sometimes it’s difficult to obtain enough sample from early colostrum, other than that the system is perfect”
- “I helped a mother several times to get out from the mess of low milk supply when receiving borderline reads by the system. We introduced more pumping sessions, and then longer pumping session, and I think that if we wouldn’t use the system early we might have needed to work much harder in order to sustain milk supply requirements. For a new mom that is already pumping and tube feeding around the clock this is super critical”
- “It gives feedback to the mother on her status, and feedback to me how to manage her case”.
- “Great tool that support my evaluation with the mother, and it help me to create a more accurate work plan”.
- “It is science fiction that become a reality!”
- “It reassures my hunches”
- “It even senses condition before I can see it, and we can start to work on milk supply earlier”
- “It gives a numeric indication to the mother, and helps her to see progress”
- “It gives validity to my work as a lactation consultant! I feel that by using the system I can give better support”.
- “The tool targets the basic concerns of any new mother, and it give them reassurance regarding their own milk supply progress, or reflects the actual state to work with”
- “I love that it strengthens my observation
