# Supplementary material for: Novel At-Home Mother’s Milk Conductivity Sensing Technology as an Identification System of Delay in Milk Secretory Activation Progress and Early Breastfeeding Problems: Feasibility Assessment
Source: JMIR Pediatr Parent. 2023 Aug 22;6:e43837. doi: 10.2196/43837 (PMC10481223; doi:10.2196/43837)
Supplement: Multimedia Appendix 3 [file pediatrics_v6i1e43837_app3.pdf]

Feasibility of a novel milk conductivity sensing device and App for assessing individual mother's milk secretory activation progress, at home

PROBLEM

Assessing and managing early **breastfeeding** and mother's Milk supply **challenges at home**

A NOVEL SYSTEM

Smartphone operated, handheld **milk sensing system** designed for at - home use with small milk sample (0.2-0.5ml) for Instant computation of a milk maturation percent parameter (**MM%**)

Remote data gather including breastfeeding and mother-baby data

Laboratory evaluation of system performance

System verified for accuracy & precision

System's output **strongly correlated to milk Na+** biomarker for secretory activation

Retrospective analysis of real-world data

Data Analyzed

30 Lactation consultants using the system in face-to-face home visits with mothers (N=555)

Mothers (N=37) Directly used the system postpartum for repeated self home-tracking

1511 milk scanning records, from real world mothers (n=592)

Retrospective Data classification

3 Groups **categorized based on reported breastfeeding status** (exclusivity & problems): 'Normal', 'Low milk supply' 'breastfeeding problems'

% Milk maturation parameter distribution show **typical time dependent** increase followed by a plateau in early postpartum period

Per-case dynamic progresses tracked at home by individual mothers

System generated **Milk maturation levels were sensitive to breastfeeding status classification**

Summary: Smart milk conductivity sensing technology enables remote data gathering and provides an appealing measure of individual breastfeeding efficiency and progress, potentially supporting self and remote care.

Haramati S, Firsow A, Navarro AD, Shechter R, Novel at-Home mother's milk conductivity sensing technology as an identification system of delay in milk secretory activation progress and early breastfeeding problems: feasibility assessment.

JMIR Publications
Advancing Digital Health & Open Science
